# Pre-eclampsia and maternal–fetal conflict

**DOI:** 10.1093/emph/eoy029

**Published:** 2018-09-12

**Authors:** P J Varas Enriquez, L J McKerracher, M G Elliot

**Affiliations:** 1 Faculty of Science, University of Amsterdam, Amsterdam, The Netherlands; 2 Department of Biochemistry and Biomedical Sciences, McMaster University, Hamilton, ON, Canada; 3 Groningen Institute for Evolutionary Life Sciences, University of Groningen, Groningen, The Netherlands

## PRE-ECLAMPSIA

Pre-eclampsia, a pregnancy disorder defined clinically by maternal hypertension and proteinuria, affects 5–10% of pregnancies and is a major cause of perinatal morbidity and mortality [1].

In healthy pregnancy, placental tissue invades maternal uterine arteries causing reduced arterial elasticity and promoting blood supply to the placenta. In early-onset pre-eclampsia (<34 weeks) this invasion is inadequate, resulting in stressed placentas deprived of blood flow and often yielding growth-restricted fetuses. In late-onset pre-eclampsia (≥34 weeks) the condition is characterized more by defects in maternal cardiovascular health than by inadequate placental invasion. In both cases, the results are a pro-inflammatory maternal immune environment, hypertension and blood vessel damage in kidney, liver and brain [2].

The multiple risk factors of pre-eclampsia include poor maternal cardiovascular health (including hypertension and obesity), presence of risk alleles and lack of extended sexual exposure of the mother to the father prior to conception [3].

## EVOLUTIONARY PERSPECTIVES

Pre-eclampsia is common only in humans (which have the most invasive form of mammalian placenta). It is reported rarely in other great apes and absent in non-apes.

Parent-offspring conflict [4] may underlie the evolutionary maintenance of pre-eclampsia despite its burden of mortality. Increased placental invasion and intimacy of maternal–fetal contact is associated with increased prenatal growth rates (but not absolute size) of brain and body [5, 6]. Since mothers and offspring have different optimal gestation lengths and growth rates, there will be conflict over these phenotypes. One additional benefit to the fetus of invasive placentation is that the metabolic costs of detoxifying harmful byproducts of oxygen metabolism (i.e. oxidative stress) can be offloaded onto the mother by direct secretion of pro-inflammatory substances into her blood [7].

Consequently, alleles promoting or suppressing invasion (of benefit to fetus and mother, respectively) persist in the population and may cause disease in certain genetic combinations or maternal health states. Conflict over placental invasion may be exacerbated by high human prenatal brain growth rates which, along with the unusually long human gestation length, may explain pre-eclampsia’s high frequency in humans but rarity in species with less-demanding fetal brain growth trajectories.

Humans have unusually low fecundability, which promotes extended preconception interaction between couples, potentially reducing pre-eclampsia risk through development of maternal tolerance of paternal antigens [3].

## FUTURE IMPLICATIONS

Genetic conflict should be expected in all pregnancies and may lead to medical complications. It may be of psychological benefit for expectant parents to anticipate conflict and its potential consequences from the beginning of pregnancy, instead of viewing pregnancy as maternal–fetal harmony.

Many mammals exhibit drastically reduced placental invasion in normal pregnancy. Understanding how these species combine low placental invasion with fetal and maternal wellbeing, and how evolutionary transitions between placental types have occurred [7], may contribute to our knowledge of human placental disorders.
